# Cytotoxic CD4 T Cells: Differentiation, Function, and Application to Dengue Virus Infection

**DOI:** 10.3389/fimmu.2016.00531

**Published:** 2016-12-07

**Authors:** Yuan Tian, Alessandro Sette, Daniela Weiskopf

**Affiliations:** ^1^Division of Vaccine Discovery, La Jolla Institute for Allergy and Immunology, La Jolla, CA, USA

**Keywords:** CD4 T cells, cytotoxicity, dengue virus, protection, vaccines

## Abstract

Dengue virus (DENV) has spread through most tropical and subtropical areas of the world and represents a serious public health problem. The control of DENV infection has not yet been fully successful due to lack of effective therapeutics or vaccines. Nevertheless, a better understanding of the immune responses against DENV infection may reveal new strategies for eliciting and improving antiviral immunity. T cells provide protective immunity against various viral infections by generating effector cells that cooperate to eliminate antigens and memory cells that can survive for long periods with enhanced abilities to control recurring pathogens. Following activation, CD8 T cells can migrate to sites of infection and kill infected cells, whereas CD4 T cells contribute to the elimination of pathogens by trafficking to infected tissues and providing help to innate immune responses, B cells, as well as CD8 T cells. However, it is now evident that CD4 T cells can also perform cytotoxic functions and induce the apoptosis of target cells. Importantly, accumulating studies demonstrate that cytotoxic CD4 T cells develop following DENV infections and may play a crucial role in protecting the host from severe dengue disease. We review our current understanding of the differentiation and function of cytotoxic CD4 T cells, with a focus on DENV infection, and discuss the potential of harnessing these cells for the prevention and treatment of DENV infection and disease.

## DENV Infection and a Protective Role for Cytotoxic CD4 T Cells

Dengue virus (DENV) is a major public health problem in tropical and subtropical areas with 390 million estimated infections per year (1). DENV has four serotypes (DENV 1–4), and infection with one of the serotypes can be asymptomatic or result in a range of diseases spanning from dengue fever (DF) to dengue hemorrhagic fever (DHF) and dengue shock syndrome (DSS). The more severe forms of DHF and DSS are more likely to develop following secondary infections with a different serotype (2). Other than supportive care there is currently no specific therapy available for the treatment of dengue diseases. Tremendous efforts have been devoted to the development of DENV vaccines since Word War II, and a tetravalent chimeric vaccine, Dengvaxia^®^, has recently been licensed in several countries including Mexico, Brazil, and the Philippines (3). However, several clinical trials of Dengvaxia^®^ raise concerns about the efficacy of the vaccine. A phase 2b study in Thailand showed an overall efficacy of 30.2% with only 9.2% protection against DENV 2 (4). Additionally, two large-scale phase 3 trials in Asia and Latin America reported that the average efficacies against the four serotypes were 56.5 and 60.8%, respectively, and further confirmed the lowest level of protection against DENV 2 (5, 6). Furthermore, a long-term follow up of these trials reveals that Dengvaxia^®^ was less effective in seronegative vaccinees and resulted in an increased incidence of hospitalization among vaccinated children who were under 9 years old (7). Given the importance of host immunity in the protection of DENV infection, it is crucial to gain a better understanding of anti-DENV immune responses and identify the correlates of protection or susceptibility in order to improve the development of immunotherapies and vaccines for dengue disease.

T cells play important roles in fighting infections with intracellular pathogens; however, the roles of T cells during DENV infection may be complex. Although some studies suggest that T cells may contribute to the pathogenesis of DENV infection *via* the production of inflammatory cytokines, and that the expansion of preexisting cross-reactive memory T cells may impair viral control upon secondary heterologous infections (original antigenic sin), others indicate that T cells may play important roles in the protection against severe dengue disease (8). Stronger T cell responses generated following natural infection or vaccination with DENV as measured by the production of effector cytokines such as interferon-γ (IFN-γ) have been associated with better protection against subsequent DENV infection (9, 10). Additionally, our laboratory has demonstrated that protective human leukocyte antigen (HLA) alleles against dengue disease are associated with robust and polyfunctional CD8 T cell responses (11). Furthermore, the observation that the frequency of T cells that express CD107a, a degranulation marker, correlates with less severe dengue disease, supporting the notion that the roles of T cells during DENV infection may depend upon their functionality and that T cells with cytotoxic potentials may be crucial for the control of DENV infection (12).

Although cytotoxic functions are usually associated with CD8 T cells, accumulating evidence has demonstrated that a range of other cells can elicit cytotoxic effector functions. Dendritic cells (DCs) are the early, primary targets of DENV in natural infection, and the vigor of cell-mediated immunity is modulated by the relative presence or absence of IFN-γ in the microenvironment surrounding the virus-infected DCs (13). DCs including Langerhans cells (LCs) express CD1d, a molecule responsible not only for the presentation of lipopeptides but also conventional antigens that have a specific binding motif, i.e., hydrophobic amino acids in position 1, 4, and 7 (14). CD1d-restricted natural killer T (NKT) cells are a distinct subset of T cells that rapidly produce an array of cytokines upon activation and play a critical role in regulating various immune responses. NKT cells are classified into two groups based on differences in T-cell receptor usage. Type I NKT cells have an invariant T-cell receptor α-chain (iNKT), while Type II NKT cells have a more diverse T-cell receptor repertoire, and it has been shown that CD4 engagement by CD1d potentiates activation of CD4^+^ NKT cells (15, 16). Recent evidence suggests iNKT involvement in DENV pathogenesis, and the level of iNKT cell activation associates with the disease severity (17–19). Finally, another unconventional T cell subset, gamma delta (γδ) T cells, has been shown to be able to kill dengue-infected cells and contribute to the immune response during DENV infection by providing an early source of IFN-γ (20).

This review focuses on CD4 T cells that can also acquire a cytotoxic phenotype, which has been investigated by numerous studies over the past three decades (21). The ability of CD4 T cells to acquire cytotoxic functions have been mostly attributed to T helper type 1 (Th1) cells after viral infections; however, it is now clear that other CD4 T cell subsets including regulatory T (Treg) cells can also secrete effector molecules and exert cytotoxic effects (22, 23). Moreover, recent studies further suggest that cytotoxic CD4 T cells may represent a separate lineage independent of other CD4 T cell subsets and are induced by distinct environmental cues and transcriptional regulators, highlighting the versatility of CD4 T cell responses (24–26). Notably, cytotoxic CD4 T cells are readily detectable following DENV infection and correlate with enhanced protection against dengue disease (12, 27). We discuss the differentiation and function of cytotoxic CD4 T cells, especially in the context of DENV infection, and anticipate future studies into the therapeutic potentials of these intriguing cells in the development of anti-DENV vaccines and immunotherapies.

## Cellular and Environmental Factors That Mediate the Generation of Cytotoxic CD4 T Cells

The differentiation of diverse CD4 T cell subsets is induced and guided by antigens, costimulation, and distinct sets of cytokines, which are integrated to regulate the expression of transcription factors that are crucial for CD4 T cell lineage specification (28). In line with this notion, costimulatory signals mediated by OX40–OX40L and 4-1BB (29–31), as well as cytokines such as transforming growth factor-β (TGF-β), type I interferons and IL-2 (25, 32–34), have been suggested to promote the differentiation of cytotoxic CD4 T cells. Although cytotoxic CD4 T cells are often observed during chronic infections such as HIV, Epstein–Barr virus (EBV), human cytomegalovirus (HCMV), and mouse CMV (MCMV) infections (35–39), they are also readily detectable following acute lymphocytic choriomeningitis virus (LCMV), influenza virus, and ectromelia virus infections (40–44). Therefore, persistent antigenic stimulation may not be absolutely required for cytotoxic CD4 T cell differentiation. Furthermore, Brown et al. reported that IL-2 substantially enhances the cytotoxic functions of CD4 T cells that are activated with low antigen dose *in vitro*, suggesting that inflammatory cytokines may amplify T cell receptor (TCR) signals to promote the differentiation of cytotoxic CD4 T cells (32). Two additional common cytokine receptor γ-chain (γ_c_) family cytokines, IL-7 and IL-15, however, are dispensable for the formation of cytotoxic CD4 T cells, although IL-15 may promote their effector functions upon reactivation with TCR stimulus (24). Interestingly, IL-21, which is another member of the γ_c_ family, has been shown to increase the production of the cytotoxic molecule granzyme B in CD8 T cells both *in vivo* and *in vitro* (45, 46). Thus, it would be interesting to investigate whether IL-21 also plays a role in the generation and/or functional maturation of cytotoxic CD4 T cells.

## Molecular Regulation of Cytotoxic CD4 T Cell Differentiation

**Figure 1:**
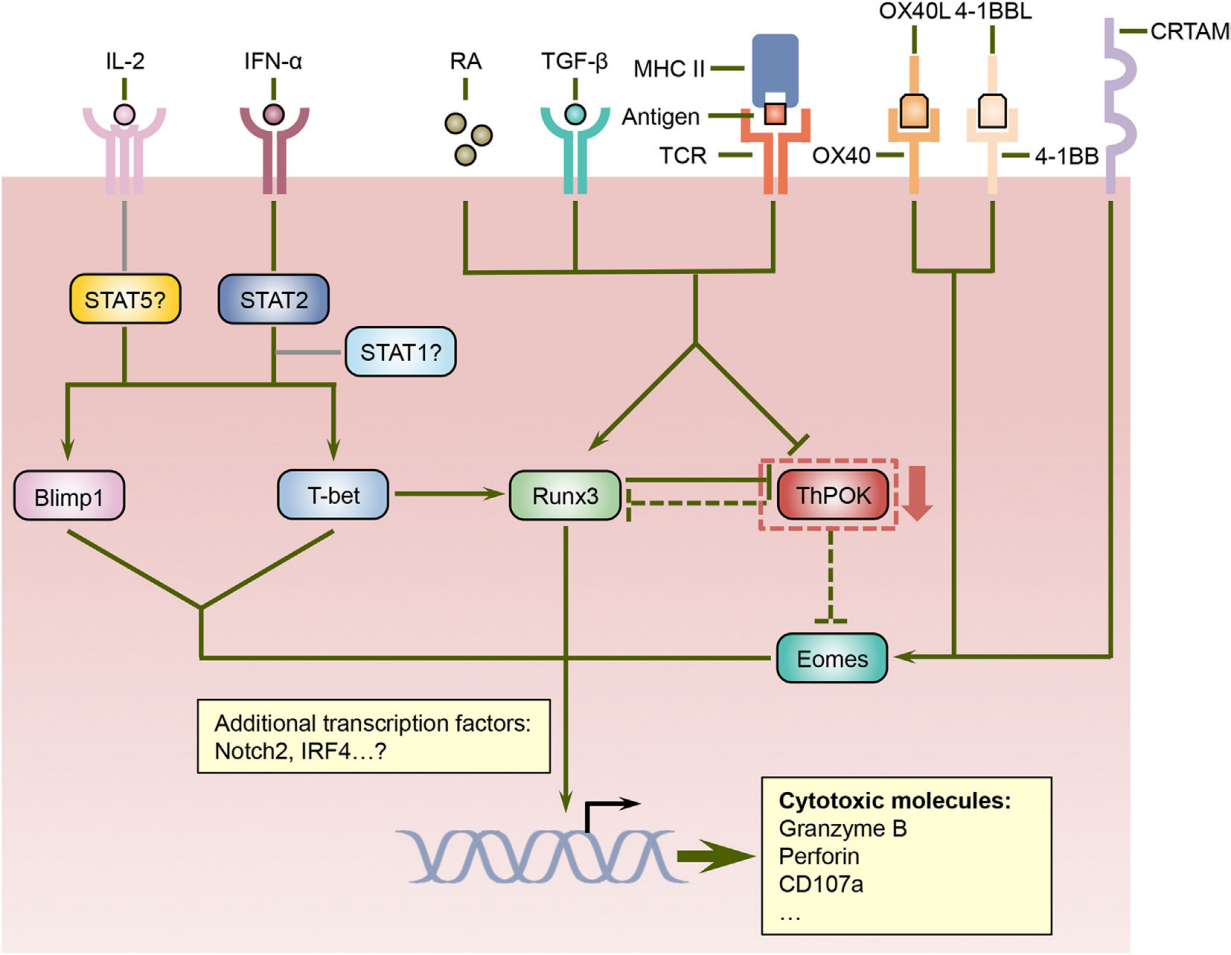
**Regulation of cytotoxic CD4 T cell differentiation by a network of signaling and transcriptional pathways**. ThPOK is essential for the lineage specification and stability of CD4 helper T cells and counteracts Runx3, which coordinate the differentiation of CD8 T cells. Nevertheless, signals mediated by antigens, TGF-β, and retinoic acid (RA) can repress ThPOK expression in CD4 T cells, which results in elevated levels of Runx3. Since ThPOK inhibits Eomes, the suppression of ThPOK may also lead to increased Eomes expression. Furthermore, cytokines such as IL-2 and IFN-α, costimulatory molecules including OX40 and 4-1BB, as well as the transmembrane protein CRTAM can increase the expression and/or activities of Eomes as well as additional transcription factors such as Blimp1 and T-bet, which together with Runx3 direct the differentiation program of cytotoxic CD4 T cells.

The integration and interpretation of numerous cellular and environmental parameters are mediated by transcriptional factors, and a number of transcriptional regulators have been implicated in the differentiation program of cytotoxic CD4 T cells (Figure 1). T-helper-inducing POZ/Kruppel-like factor (ThPOK) and Runt-related transcription factor 3 (Runx3), which suppress each other’s expression, control the development of CD4 and CD8 T cells in the thymus, respectively (47). After exiting the thymus, mature CD4 T cells continue to express ThPOK, which suppresses Runx3 and maintains the lineage stability of CD4 T cells (48–50). Ectopic expression of ThPOK in CD8 T cells results in reduced expression of CD8, the T-box transcription factor eomesodermin (Eomes), as well as effector molecules such as IFN-γ, granzyme B, and perforin, further supporting the notion that ThPOK restricts the initiation of cytotoxic T lymphocyte (CTL) differentiation program in CD4 T cells (51). In contrast, Runx3 promotes CD8 expression by binding its enhancer regions (52, 53) and also cooperates with Eomes and another T-box transcription factor, T-bet, to induce the manufacture of IFN-γ, perforin, and granzyme B (54, 55). Intriguingly, a portion of CD4 T cells downregulates their expression of ThPOK in the intestine, especially in the intraepithelial lymphocyte (IEL) compartment, under unimmunized conditions or following activation with their cognate antigen (24, 25). Conversely, these ThPOK^low^ CD4 T cells upregulate the expression of Runx3, thus resembling their CD8 T cell counterparts (24, 25). Consequently, these cells showed enhanced expression of cytotoxic effector lymphocytes-associated molecules, including 2B4, granzyme B, and CD107a, and demonstrated cytotoxicity *in vitro* (24, 25). It is further proposed that sustained antigenic stimulation and/or local environmental cues such as TGF-β and retinoic acid (RA) induce the downregulation of ThPOK and the upregulation of Runx3, although the underlying signaling and transcriptional mechanisms are less well defined (24, 25). Thus, the antagonistic expression of ThPOK and Runx3 not only dictate the lineage stability of CD4 and CD8 T cells but also direct the establishment of cytotoxic CD4 T cells. Further studies are needed to determine whether and how the expression of ThPOK and Runx3 dictate the development of cytotoxic CD4 T cells following viral infections as well as the cellular and environmental factors that modulate their expression.

In addition to ThPOK and Runx3, several other transcription regulators have been suggested to regulate the differentiation of cytotoxic CD4 T cells. T-bet promotes the differentiation of effector CD8 T cells (56) and also induces the expression of Runx3 in CD4 T cells (54), suggesting that it may play a role in the formation of cytotoxic CD4 T cells. Indeed, T-bet-deficient CD4 T cells demonstrated substantially reduced production of granzyme B following influenza virus infection (33). Furthermore, the acquisition of cytotoxic functions by CD4 T cells is also dependent upon B lymphocyte-induced maturation protein 1 (Blimp1), which facilitates the binding of T-bet to the granzyme B and perforin promoters (33). The upstream signals that drive the expression of T-bet and Blimp1 in CD4 T cells include IL-2 and IFN-α, and IFN-α may also exert its effects partially *via* signal transducer and activator of transcription 2 (STAT2) as evidenced by decreased T-bet and granzyme B expression in the absence of STAT2 (33). Since heterodimerization with activated STAT1 is required for the nuclear accumulation of phosphorylated STAT2 (57), whether STAT1 promotes cytotoxic CD4 T cell development in conjunction with STAT2 warrants further investigation. Surprisingly, STAT4, which has been shown to promote T-bet expression and Th1 cell differentiation in response to IL-12 signals (58–60), is dispensable for the upregulation of T-bet and granzyme B in CD4 T cells following influenza virus infection (33), indicating that cytotoxic CD4 T cells and Th1 cells may rely on distinct pathways for their differentiation. As discussed above, ThPOK suppresses the expression of Eomes, which cooperates with T-bet and Runx3 to promote the effector functions of CD8 T cells (55, 61–63), suggesting that Eomes may also participate in programing cytotoxic CD4 T cells when its expression is increased. Indeed, the engagement of costimulatory molecules OX40 and/or 4-1BB induces the expression of Eomes, which then upregulates the production of granzyme B by CD4 T cells and enhances their antitumor activities (29–31). In addition, a recent report shows that class I-restricted T cell-associated molecule (CRTAM) can promote the expression of Eomes and cytotoxic proteins including granzyme B and perforin in CD4 T cells (64). Therefore, Eomes may coordinate multiple signaling pathways to direct the development of cytotoxic CD4 T cells. Additional transcriptional regulators such as Notch2 (65), STAT5 (66), and interferon regulatory factor 4 (IRF4) (67) have been implicated in the manufacture of cytotoxic weaponry in CD8 T cells, and it would be interesting to investigate whether these transcriptional regulators also modulate the cytotoxic potential of CD4 T cells. In sum, the signals mediated by TCR, cytokines, costimulatory molecules, and other cell surface receptors are integrated and interpreted by a network of transcriptional regulators, which collectively orchestrate the differentiation of cytotoxic CD4 T cells.

## The Development of Cytotoxic CD4 T Cells Following DENV Infections

DENV-specific CD4 T cells with cytotoxic potential were initially observed with T cell clones isolated from a DENV-infected donor (68). These CD4 T cell clones demonstrate *in vitro* killing of target cells that display DENV antigens as assessed by chromium release assays, which are restricted by HLA class II molecules (68). Subsequent studies discovered that DENV non-structural (NS) proteins especially NS3 are the major targets of cytotoxic CD4 T cell clones and that many of these cell clones exhibit cross-reactivity against several DENV serotypes (69–72). Thus, cytotoxic CD4 T cell may preferentially recognize conserved DENV antigens, and repeated antigenic stimulation may favor their formation. In addition to antigen-specific killing, anti-DENV cytotoxic CD4 T cell clones generated from DENV-immune donor can also mediate the lysis of non-antigen-presenting bystander target cells (73). While cytotoxic CD4 T cells lyse target cells pulsed with DENV antigens primarily *via* perforin-dependent mechanisms, the lysis of bystander target cells mainly relies on the Fas/Fas ligand (FasL) pathway (73). Additionally, a cytotoxic CD4 T cell clone has also been isolated from DENV-infected mice and is able to kill DENV antigen-pulsed target cells *in vitro* (74). Furthermore, Yauch et al. showed, using a mouse model of DENV infection, that CD4 T cells can mediate DENV-specific killing of target cells *in vivo*, although the production of cytotoxic molecules by CD4 T cells was not assessed (75). Immunization with CD4 T cell epitopes derived from DENV NS proteins NS2B and NS3 can accelerate viral clearance following DENV challenge, suggesting that the induction of cytotoxic CD4 T cells by vaccination may be beneficial for the control of secondary infections with DENV (75).

Previous studies have demonstrated that CD4 T cells with cytotoxic potential as assessed by the expression of CD107a are present in patients associated with both primary and secondary DENV infections, although the frequencies of these cells vary according to infection history and disease severity (12). Interestingly, the frequency of DENV-specific CD107a^+^ CD4 T cells is higher in DF patients compared with those who had a more severe form of the disease, DHF, implicating a protective role for cytotoxic CD4 T cells in DENV-infected patients (12). Our laboratory has recently discovered that a subset of CD4 T cells expand as a function of DENV infection history and is most prominently represented in donors associated with multiple DENV infections (27). These CD4 T cells display a CD45RA^high^CCR7^low^ phenotype, which is distinct from their CD45RA^low^CCR7^high^ central memory T (Tcm) and CD45RA^low^CCR7^low^ effector memory T (Tem) counterparts, and thus are designated effector memory RA T (Temra) cells. Compared with CD4 Tcm or Tem cells, a higher proportion of CD4 Temra cells express CD8α, the degranulation marker CD107a, as well as other cytotoxic effector molecules such as granzyme B and perforin, suggesting that CD4 Temra population contains anti-DENV cytotoxic CD4 T cells (27). Additionally, CD4 Temra cells also had increased expression of CD226 (27), which is a costimulatory molecule that has been shown to enhance the effector and cytotoxic functions of CD8 T cells (76, 77). Conversely, these cells downregulate CD28 expression (19), which is consistent with previous observations that CD4^+^CD28^−^ T cells are associated with enhanced cytotoxic functions following infections with CMV and hepatitis B virus (HBV) (78, 79). As discussed earlier, the T-box transcription factors T-bet and Eomes coordinate the development of cytotoxic CD4 T cells. Notably, the vast majority of CD4 Temra cells express high levels of T-bet and Eomes, further supporting the notion that CD4 Temra cells encompass cytotoxic CD4 T cell subset in terms of their phenotypic and functional attributes, as well as their transcriptional signatures. Since cytotoxic CD4 Temra cells are generally detected following secondary DENV infections, these findings further support that DENV-specific cytotoxic CD4 T cells are induced by repeated TCR stimulation from conserved DENV antigens, which is consistent with *in vitro* studies using cytotoxic CD4 T cell clones. Intriguingly, CD4 Temra cells with cytotoxic potential observed in secondary DENV-infected donors phenotypically and functionally resemble the live attenuated yellow fever vaccine 17D (YF-17D)-elicited CD8 Temra cells, which are highly proliferative and polyfunctional as evidenced by their ability to produce various cytokines and cytotoxic molecules including CD107a (80). Since vaccination with YF-17D has been tremendously successful in controlling yellow fever virus (81), Temra phenotype cytotoxic CD4 T cells may be highly relevant in vaccine-elicited protection against DENV infection.

The majority of DENV-specific CD4 Temra cells are not associated with the phenotypes of Th1, Th2, or Th17 cells as evidenced by their lack of expression of the chemokine receptors CXCR3, CCR4, and CCR6, which have been used to distinguish between these distinct CD4 T cell subsets (27, 82). Since CD4 Temra cells encompass cytotoxic populations, these findings further support the notion that cytotoxic CD4 T cells may represent an independent CD4 T cell lineage. Notably, Temra cells upregulate the expression of the chemokine receptor CX3CR1, which binds to CX3CL1 (fractalkine) and has been implicated in promoting the adhesion and migration of CD8 T cells (83, 84). Thus, CX3CR1 may facilitate the trafficking of DENV-specific CD4 T cells, particularly cytotoxic CD4 T cells, to infected tissues. Intriguingly, accumulating studies have demonstrated that CD4 tissue-resident memory T (Trm) cells reside in sites of pathogen entry and are crucial for the control of viral pathogens such as influenza virus and herpes simplex virus by providing immediate effector functions (85, 86). Therefore, it would be interesting to investigate whether DENV-specific CD4 Trm cells develop following infections and whether CX3CR1 plays a role in their establishment and maintenance in non-lymphoid tissues such as the skin. Importantly, the expression of CX3CR1 is also associated with the cytotoxic functions of CD4 T cells as isolated CX3CR1^+^ CD4 T cells demonstrated specific killing of DENV epitopes-pulsed target cells *ex vivo* (27). This is consistent with a recent report showing that CX3CR1^+^ CD8 T cells have potent cytotoxic functions and express elevated levels of granzyme B and perforin (87). Thus, CX3CR1^+^ cytotoxic CD4 T cells may play an important role in viral control by directly targeting DENV-infected cells in tissues.

## Cytotoxic CD4 T Cells Correlate with HLA-Associated Protection Against DENV Infection

**Figure 2:**
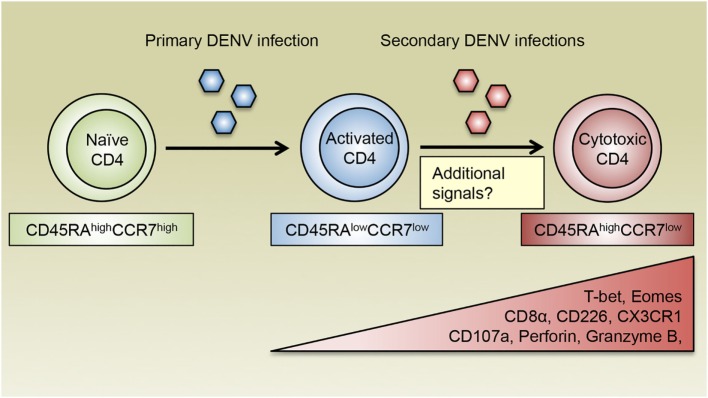
**The generation of cytotoxic CD4 T cells following DENV infections**. DENV-specific naïve CD4 T cells are activated during primary DENV infection and acquire an effector phenotype and the ability to produce inflammatory cytokines such as IFN-γ. Following reexposure to secondary heterologous infections, DENV-specific CD4 T cells receive repeated antigenic signals and differentiate into cytotoxic CD4 T cells, which display a CD45RA^high^CCR7^low^ Temra phenotype and are characterized by their expression of the chemokine receptor CX3CR1. Cytotoxic CD4 T cells also upregulate the expression of CD8α and CD226, as well as the transcription factors T-bet and Eomes, which may cooperate with additional transcription regulators to induce the production of cytotoxic molecules such as CD107a, perforin, and granzyme B. In addition to recurring antigens, costimulatory molecules, cytokines, and other environmental cues are all likely to modulate the differentiation of cytotoxic CD4 T cells.

Additional insights into the question of whether cytotoxic CD4 T cells are beneficial in the context of DENV infections come from the association of HLA allelic variants with relative susceptibility or resistance to severe DENV-associated diseases. For instance, HLA class II molecules DRB1*04:01 and DRB1*08:02 are associated with resistance and susceptibility to severe DENV diseases, respectively (88–90). Intriguingly, the frequency of DENV-specific CD4 Temra cells is higher in donors expressing the protective allele DRB1*04:01 compared with those expressing the susceptible allele DRB1*08:02. Moreover, CD4 Temra cells restricted by DRB1*04:01 express substantially higher levels of cytotoxic proteins including CD107a, perforin, and granzyme B compared with their DRB1*08:02-restricted counterparts. Thus, increased abundance and functionality of cytotoxic CD4 Temra cells may be associated with enhanced protection against severe dengue disease. Furthermore, the capacity of CX3CR1^+^ CD4 T cells to kill target cells varies according to HLA restrictions with the protective allele DRB1*04:01-restricted cells showing higher cytotoxic activities than their susceptible allele DRB1*08:02-restricted counterparts, which again indicates that cytotoxic CD4 T cell responses correlate with protection from severe dengue disease. Since DENV primarily infects major histocompatibility complex (MHC) class II-expressing antigen-presenting cells such as monocytes, macrophages, and DCs (91, 92), which could be exacerbated by antibody-dependent enhancement (ADE) mechanism (93), cytotoxic CD4 T cells may play important roles in controlling the spread of DENV infection by directly eliminating these cells. Other cell types such as epithelial cells can be induced to upregulate the expression of MHC class II molecules following viral infection and potentially become additional targets for cytotoxic CD4 T cells (43). Therefore, cytotoxic CD4 T cells may contribute to HLA-associated protection against DENV infection by targeting DENV-infected cells of various types. Taken together, we propose a model where repeated antigenic signals as well as other potential cellular and environmental stimuli facilitate the formation of DENV-specific cytotoxic CD4 T cells, which exhibit a Temra phenotype with upregulated CX3CR1 expression and mediate protective responses against DENV infection (Figure 2).

## Conclusion and Perspective

Despite the recent approval of Dengvaxia^®^ in several countries where DENV is epidemic, our need for an efficacious DENV vaccine is still unsatisfied. Accumulating studies strongly indicate that, in addition to CD8 T cells, cytotoxic CD4 T cells may play an important role in eliminating DENV-infected cells and protecting the hosts from severe dengue disease. Since the generation of cytotoxic CD4 T cells is concurrent with multiple DENV infections, it would be interesting to identify the TCR-specificity of cytotoxic CD4 T cells and confirm whether they respond to conserved epitopes shared by different DENV serotypes. This would allow for the design of vaccines that include such epitopes and preferentially induce the formation of cytotoxic CD4 T cells. In addition to vaccines, adoptive transfer of engineered CD4 T cells that are specific for such antigens may accelerate viral clearance and benefit the treatment of DENV infection, as clinical trials of T cells that are engineered to express transgenic TCRs or chimeric antigen receptors (CARs) have generated promising results in treating cancers (94). Furthermore, costimulatory molecules and cytokines that are involved in the differentiation and function of cytotoxic CD4 T cells may potentially be used as adjuvants to enhance the cytotoxic effects of DENV-specific CD4 T cells. Additionally, elucidating the factors that control the development of cytotoxic CD4 T cells may allow one to manipulate their expression, availability, and activity in order to reshape CD4 T cell responses in patients expressing susceptible alleles and redirect antiviral CD4 T cells to differentiate into potent cytotoxic cells. Finally, T cell differentiation and function is greatly influenced by nutrients and metabolism (95). For instance, glucose and glycolysis promote the expression of cytotoxic molecules in CD8 T cells (96). Thus, modulation of the metabolic program may provide additional opportunities to enhance cytotoxic CD4 T cell response. In summary, future investigations into the antigenic, environmental, and cellular parameters that configure the formation, migration, and maintenance of cytotoxic CD4 T cells may reveal novel strategies for developing and improving vaccines and therapies that fight DENV as well as other emerging pathogens.

## Author Contributions

YT wrote the manuscript; YT and DW designed the figures; AS and DW critically edited the manuscript.

## Conflict of Interest Statement

The authors declare that the research was conducted in the absence of any commercial or financial relationships that could be construed as a potential conflict of interest.

## References

[1] Bhatt S Gething PW Brady OJ Messina JP Farlow AW Moyes CL The global distribution and burden of dengue. Nature (2013) 496:504–7. 10.1038/nature12060 23563266 PMC3651993

[2] Halstead SB. Pathogenesis of dengue: challenges to molecular biology. Science (1988) 239:476–81. 10.1126/science.3277268 3277268

[3] Alagarasu K. Introducing dengue vaccine: implications for diagnosis in dengue vaccinated subjects. Vaccine (2016) 34:2759–61. 10.1016/j.vaccine.2016.04.070 27142330

[4] Sabchareon A Wallace D Sirivichayakul C Limkittikul K Chanthavanich P Suvannadabba S Protective efficacy of the recombinant, live-attenuated, CYD tetravalent dengue vaccine in Thai schoolchildren: a randomised, controlled phase 2b trial. Lancet (2012) 380:1559–67. 10.1016/S0140-6736(12)61428-7 22975340

[5] Villar L Dayan GH Arredondo-Garcia JL Rivera DM Cunha R Deseda C Efficacy of a tetravalent dengue vaccine in children in Latin America. N Engl J Med (2015) 372:113–23. 10.1056/NEJMoa1411037 25365753

[6] Capeding MR Tran NH Hadinegoro SR Ismail HI Chotpitayasunondh T Chua MN Clinical efficacy and safety of a novel tetravalent dengue vaccine in healthy children in Asia: a phase 3, randomised, observer-masked, placebo-controlled trial. Lancet (2014) 384:1358–65. 10.1016/S0140-6736(14)61060-6 25018116

[7] Hadinegoro SR Arredondo-Garcia JL Capeding MR Deseda C Chotpitayasunondh T Dietze R Efficacy and long-term safety of a dengue vaccine in regions of endemic disease. N Engl J Med (2015) 373:1195–206. 10.1056/NEJMoa1506223 26214039

[8] Weiskopf D Sette A. T-cell immunity to infection with dengue virus in humans. Front Immunol (2014) 5:93. 10.3389/fimmu.2014.00093 24639680 PMC3945531

[9] Gunther VJ Putnak R Eckels KH Mammen MP Scherer JM Lyons A A human challenge model for dengue infection reveals a possible protective role for sustained interferon gamma levels during the acute phase of illness. Vaccine (2011) 29:3895–904. 10.1016/j.vaccine.2011.03.038 21443963

[10] Hatch S Endy TP Thomas S Mathew A Potts J Pazoles P Intracellular cytokine production by dengue virus-specific T cells correlates with subclinical secondary infection. J Infect Dis (2011) 203:1282–91. 10.1093/infdis/jir012 21335561 PMC3069729

[11] Weiskopf D Angelo MA de Azeredo EL Sidney J Greenbaum JA Fernando AN Comprehensive analysis of dengue virus-specific responses supports an HLA-linked protective role for CD8+ T cells. Proc Natl Acad Sci U S A (2013) 110:E2046–53. 10.1073/pnas.1305227110 23580623 PMC3670335

[12] Duangchinda T Dejnirattisai W Vasanawathana S Limpitikul W Tangthawornchaikul N Malasit P Immunodominant T-cell responses to dengue virus NS3 are associated with DHF. Proc Natl Acad Sci U S A (2010) 107:16922–7. 10.1073/pnas.1010867107 20837518 PMC2947904

[13] Libraty DH Pichyangkul S Ajariyakhajorn C Endy TP Ennis FA. Human dendritic cells are activated by dengue virus infection: enhancement by gamma interferon and implications for disease pathogenesis. J Virol (2001) 75:3501–8. 10.1128/JVI.75.8.3501-3508.2001 11264339 PMC114841

[14] Bendelac A CD1: presenting unusual antigens to unusual T lymphocytes. Science (1995) 269:185–6. 10.1126/science.7542402 7542402

[15] Liao CM Zimmer MI Wang CR. The functions of type I and type II natural killer T cells in inflammatory bowel diseases. Inflamm Bowel Dis (2013) 19:1330–8. 10.1097/MIB.0b013e318280b1e3 23518808 PMC3694171

[16] Thedrez A de Lalla C Allain S Zaccagnino L Sidobre S Garavaglia C CD4 engagement by CD1d potentiates activation of CD4+ invariant NKT cells. Blood (2007) 110:251–8. 10.1182/blood-2007-01-066217 17363727

[17] Matangkasombut P Chan-In W Opasawaschai A Pongchaikul P Tangthawornchaikul N Vasanawathana S Invariant NKT cell response to dengue virus infection in human. PLoS Negl Trop Dis (2014) 8:e2955. 10.1371/journal.pntd.0002955 24945350 PMC4063705

[18] Renneson J Guabiraba R Maillet I Marques RE Ivanov S Fontaine J A detrimental role for invariant natural killer T cells in the pathogenesis of experimental dengue virus infection. Am J Pathol (2011) 179:1872–83. 10.1016/j.ajpath.2011.06.023 21843496 PMC3181339

[19] St John AL Rathore AP Yap H Ng ML Metcalfe DD Vasudevan SG Immune surveillance by mast cells during dengue infection promotes natural killer (NK) and NKT-cell recruitment and viral clearance. Proc Natl Acad Sci U S A (2011) 108:9190–5. 10.1073/pnas.1105079108 21576486 PMC3107258

[20] Tsai CY Liong KH Gunalan MG Li N Lim DS Fisher DA Type I IFNs and IL-18 regulate the antiviral response of primary human gammadelta T cells against dendritic cells infected with dengue virus. J Immunol (2015) 194:3890–900. 10.4049/jimmunol.1303343 25732728

[21] Marshall NB Swain SL. Cytotoxic CD4 T cells in antiviral immunity. J Biomed Biotechnol (2011) 2011:954602. 10.1155/2011/954602 22174559 PMC3228492

[22] Grossman WJ Verbsky JW Barchet W Colonna M Atkinson JP Ley TJ. Human T regulatory cells can use the perforin pathway to cause autologous target cell death. Immunity (2004) 21:589–601. 10.1016/j.immuni.2004.09.002 15485635

[23] Cao X Cai SF Fehniger TA Song J Collins LI Piwnica-Worms DR Granzyme B and perforin are important for regulatory T cell-mediated suppression of tumor clearance. Immunity (2007) 27:635–46. 10.1016/j.immuni.2007.08.014 17919943

[24] Mucida D Husain MM Muroi S van Wijk F Shinnakasu R Naoe Y Transcriptional reprogramming of mature CD4(+) helper T cells generates distinct MHC class II-restricted cytotoxic T lymphocytes. Nat Immunol (2013) 14:281–9. 10.1038/ni.2523 23334788 PMC3581083

[25] Reis BS Rogoz A Costa-Pinto FA Taniuchi I Mucida D Mutual expression of the transcription factors Runx3 and ThPOK regulates intestinal CD4(+) T cell immunity. Nat Immunol (2013) 14:271–80. 10.1038/ni.2518 23334789 PMC3804366

[26] Cheroutre H Husain MM. CD4 CTL: living up to the challenge. Semin Immunol (2013) 25:273–81. 10.1016/j.smim.2013.10.022 24246226 PMC3886800

[27] Weiskopf D Bangs DJ Sidney J Kolla RV De Silva AD de Silva AM Dengue virus infection elicits highly polarized CX3CR1+ cytotoxic CD4+ T cells associated with protective immunity. Proc Natl Acad Sci U S A (2015) 112:E4256–63. 10.1073/pnas.1505956112 26195744 PMC4534238

[28] Tripathi SK Lahesmaa R. Transcriptional and epigenetic regulation of T-helper lineage specification. Immunol Rev (2014) 261:62–83. 10.1111/imr.12204 25123277 PMC4255756

[29] Curran MA Geiger TL Montalvo W Kim M Reiner SL Al-Shamkhani A Systemic 4-1BB activation induces a novel T cell phenotype driven by high expression of eomesodermin. J Exp Med (2013) 210:743–55. 10.1084/jem.20121190 23547098 PMC3620352

[30] Hirschhorn-Cymerman D Budhu S Kitano S Liu C Zhao F Zhong H Induction of tumoricidal function in CD4+ T cells is associated with concomitant memory and terminally differentiated phenotype. J Exp Med (2012) 209:2113–26. 10.1084/jem.20120532 23008334 PMC3478933

[31] Qui HZ Hagymasi AT Bandyopadhyay S St Rose MC Ramanarasimhaiah R Menoret A CD134 plus CD137 dual costimulation induces eomesodermin in CD4 T cells to program cytotoxic Th1 differentiation. J Immunol (2011) 187:3555–64. 10.4049/jimmunol.1101244 21880986 PMC3178659

[32] Brown DM Kamperschroer C Dilzer AM Roberts DM Swain SL. IL-2 and antigen dose differentially regulate perforin- and FasL-mediated cytolytic activity in antigen specific CD4+ T cells. Cell Immunol (2009) 257:69–79. 10.1016/j.cellimm.2009.03.002 19338979 PMC2683476

[33] Hua L Yao S Pham D Jiang L Wright J Sawant D Cytokine-dependent induction of CD4+ T cells with cytotoxic potential during influenza virus infection. J Virol (2013) 87:11884–93. 10.1128/JVI.01461-13 23986597 PMC3807312

[34] Workman AM Jacobs AK Vogel AJ Condon S Brown DM. Inflammation enhances IL-2 driven differentiation of cytolytic CD4 T cells. PLoS One (2014) 9:e89010. 10.1371/journal.pone.0089010 24586481 PMC3930678

[35] Appay V Zaunders JJ Papagno L Sutton J Jaramillo A Waters A Characterization of CD4(+) CTLs ex vivo. J Immunol (2002) 168:5954–8. 10.4049/jimmunol.168.11.5954 12023402

[36] Casazza JP Betts MR Price DA Precopio ML Ruff LE Brenchley JM Acquisition of direct antiviral effector functions by CMV-specific CD4+ T lymphocytes with cellular maturation. J Exp Med (2006) 203:2865–77. 10.1084/jem.20052246 17158960 PMC2118179

[37] Suni MA Ghanekar SA Houck DW Maecker HT Wormsley SB Picker LJ CD4(+)CD8(dim) T lymphocytes exhibit enhanced cytokine expression, proliferation and cytotoxic activity in response to HCMV and HIV-1 antigens. Eur J Immunol (2001) 31:2512–20. 10.1002/1521-4141(200108)31:8<2512::AID-IMMU2512>3.0.CO;2-M 11500836

[38] Verma S Weiskopf D Gupta A McDonald B Peters B Sette A Cytomegalovirus-specific CD4 T cells are cytolytic and mediate vaccine protection. J Virol (2016) 90:650–8. 10.1128/JVI.02123-15 PMC4702662 26491148

[39] Haigh TA Lin X Jia H Hui EP Chan AT Rickinson AB EBV latent membrane proteins (LMPs) 1 and 2 as immunotherapeutic targets: LMP-specific CD4+ cytotoxic T cell recognition of EBV-transformed B cell lines. J Immunol (2008) 180:1643–54. 10.4049/jimmunol.180.3.1643 18209060

[40] Zajac AJ Quinn DG Cohen PL Frelinger JA. Fas-dependent CD4+ cytotoxic T-cell-mediated pathogenesis during virus infection. Proc Natl Acad Sci U S A (1996) 93:14730–5. 10.1073/pnas.93.25.14730 8962123 PMC26204

[41] Jellison ER Kim SK Welsh RM. Cutting edge: MHC class II-restricted killing in vivo during viral infection. J Immunol (2005) 174:614–8. 10.4049/jimmunol.174.2.614 15634878

[42] Brown DM Lee S Garcia-Hernandez Mde L Swain SL. Multifunctional CD4 cells expressing gamma interferon and perforin mediate protection against lethal influenza virus infection. J Virol (2012) 86:6792–803. 10.1128/JVI.07172-11 22491469 PMC3393557

[43] Wilkinson TM Li CK Chui CS Huang AK Perkins M Liebner JC Preexisting influenza-specific CD4+ T cells correlate with disease protection against influenza challenge in humans. Nat Med (2012) 18:274–80. 10.1038/nm.2612 22286307

[44] Fang M Siciliano NA Hersperger AR Roscoe F Hu A Ma X Perforin-dependent CD4+ T-cell cytotoxicity contributes to control a murine poxvirus infection. Proc Natl Acad Sci U S A (2012) 109:9983–8. 10.1073/pnas.1202143109 22665800 PMC3382508

[45] Tian Y Cox MA Kahan SM Ingram JT Bakshi RK Zajac AJ. A context-dependent role for IL-21 in modulating the differentiation, distribution, and abundance of effector and memory CD8 T cell subsets. J Immunol (2016) 196:2153–66. 10.4049/jimmunol.1401236 26826252 PMC4761492

[46] Sutherland AP Joller N Michaud M Liu SM Kuchroo VK Grusby MJ. IL-21 promotes CD8+ CTL activity via the transcription factor T-bet. J Immunol (2013) 190:3977–84. 10.4049/jimmunol.1201730 23479229

[47] Egawa T. Regulation of CD4 and CD8 coreceptor expression and CD4 versus CD8 lineage decisions. Adv Immunol (2015) 125:1–40. 10.1016/bs.ai.2014.09.001 25591463

[48] Wang L Wildt KF Castro E Xiong Y Feigenbaum L Tessarollo L The zinc finger transcription factor Zbtb7b represses CD8-lineage gene expression in peripheral CD4+ T cells. Immunity (2008) 29:876–87. 10.1016/j.immuni.2008.09.019 19062319 PMC3392968

[49] Egawa T Littman DR. ThPOK acts late in specification of the helper T cell lineage and suppresses Runx-mediated commitment to the cytotoxic T cell lineage. Nat Immunol (2008) 9:1131–9. 10.1038/ni.1652 18776905 PMC2666788

[50] Vacchio MS Wang L Bouladoux N Carpenter AC Xiong Y Williams LC A ThPOK-LRF transcriptional node maintains the integrity and effector potential of post-thymic CD4+ T cells. Nat Immunol (2014) 15:947–56. 10.1038/ni.2960 25129370 PMC4251968

[51] Jenkinson SR Intlekofer AM Sun G Feigenbaum L Reiner SL Bosselut R. Expression of the transcription factor cKrox in peripheral CD8 T cells reveals substantial postthymic plasticity in CD4-CD8 lineage differentiation. J Exp Med (2007) 204:267–72. 10.1084/jem.20061982 17296789 PMC2118724

[52] Sato T Ohno S Hayashi T Sato C Kohu K Satake M Dual functions of Runx proteins for reactivating CD8 and silencing CD4 at the commitment process into CD8 thymocytes. Immunity (2005) 22:317–28. 10.1016/j.immuni.2005.01.012 15780989

[53] Hassan H Sakaguchi S Tenno M Kopf A Boucheron N Carpenter AC Cd8 enhancer E8I and Runx factors regulate CD8alpha expression in activated CD8+ T cells. Proc Natl Acad Sci U S A (2011) 108:18330–5. 10.1073/pnas.1105835108 22025728 PMC3215065

[54] Djuretic IM Levanon D Negreanu V Groner Y Rao A Ansel KM. Transcription factors T-bet and Runx3 cooperate to activate Ifng and silence Il4 in T helper type 1 cells. Nat Immunol (2007) 8:145–53. 10.1038/ni1424 17195845

[55] Cruz-Guilloty F Pipkin ME Djuretic IM Levanon D Lotem J Lichtenheld MG Runx3 and T-box proteins cooperate to establish the transcriptional program of effector CTLs. J Exp Med (2009) 206:51–9. 10.1084/jem.20081242 19139168 PMC2626671

[56] Joshi NS Cui W Chandele A Lee HK Urso DR Hagman J Inflammation directs memory precursor and short-lived effector CD8(+) T cell fates via the graded expression of T-bet transcription factor. Immunity (2007) 27:281–95. 10.1016/j.immuni.2007.07.010 17723218 PMC2034442

[57] Banninger G Reich NC STAT2 nuclear trafficking. J Biol Chem (2004) 279:39199–206. 10.1074/jbc.M400815200 15175343

[58] Schulz EG Mariani L Radbruch A Hofer T. Sequential polarization and imprinting of type 1 T helper lymphocytes by interferon-gamma and interleukin-12. Immunity (2009) 30:673–83. 10.1016/j.immuni.2009.03.013 19409816

[59] Thieu VT Yu Q Chang HC Yeh N Nguyen ET Sehra S Signal transducer and activator of transcription 4 is required for the transcription factor T-bet to promote T helper 1 cell-fate determination. Immunity (2008) 29:679–90. 10.1016/j.immuni.2008.08.017 18993086 PMC2768040

[60] Zhu J Jankovic D Oler AJ Wei G Sharma S Hu G The transcription factor T-bet is induced by multiple pathways and prevents an endogenous Th2 cell program during Th1 cell responses. Immunity (2012) 37:660–73. 10.1016/j.immuni.2012.09.007 23041064 PMC3717271

[61] Pearce EL Mullen AC Martins GA Krawczyk CM Hutchins AS Zediak VP Control of effector CD8+ T cell function by the transcription factor eomesodermin. Science (2003) 302:1041–3. 10.1126/science.1090148 14605368

[62] Intlekofer AM Takemoto N Wherry EJ Longworth SA Northrup JT Palanivel VR Effector and memory CD8+ T cell fate coupled by T-bet and eomesodermin. Nat Immunol (2005) 6:1236–44. 10.1038/ni1268 16273099

[63] Pipkin ME Sacks JA Cruz-Guilloty F Lichtenheld MG Bevan MJ Rao A. Interleukin-2 and inflammation induce distinct transcriptional programs that promote the differentiation of effector cytolytic T cells. Immunity (2010) 32:79–90. 10.1016/j.immuni.2009.11.012 20096607 PMC2906224

[64] Takeuchi A Badr Mel S Miyauchi K Ishihara C Onishi R Guo Z CRTAM determines the CD4+ cytotoxic T lymphocyte lineage. J Exp Med (2016) 213:123–38. 10.1084/jem.20150519 26694968 PMC4710199

[65] Maekawa Y Minato Y Ishifune C Kurihara T Kitamura A Kojima H Notch2 integrates signaling by the transcription factors RBP-J and CREB1 to promote T cell cytotoxicity. Nat Immunol (2008) 9:1140–7. 10.1038/ni.1649 18724371

[66] Verdeil G Puthier D Nguyen C Schmitt-Verhulst AM Auphan-Anezin N. STAT5-mediated signals sustain a TCR-initiated gene expression program toward differentiation of CD8 T cell effectors. J Immunol (2006) 176:4834–42. 10.4049/jimmunol.176.8.4834 16585578

[67] Raczkowski F Ritter J Heesch K Schumacher V Guralnik A Hocker L The transcription factor interferon regulatory factor 4 is required for the generation of protective effector CD8+ T cells. Proc Natl Acad Sci U S A (2013) 110:15019–24. 10.1073/pnas.1309378110 23980171 PMC3773801

[68] Kurane I Meager A Ennis FA Dengue virus-specific human T cell clones. Serotype crossreactive proliferation, interferon gamma production, and cytotoxic activity. J Exp Med (1989) 170:763–75. 10.1084/jem.170.3.763 2475573 PMC2189437

[69] Kurane I Brinton MA Samson AL Ennis FA. Dengue virus-specific, human CD4+ CD8- cytotoxic T-cell clones: multiple patterns of virus cross-reactivity recognized by NS3-specific T-cell clones. J Virol (1991) 65:1823–8. 1705990 10.1128/jvi.65.4.1823-1828.1991 PMC239991

[70] Zeng L Kurane I Okamoto Y Ennis FA Brinton MA. Identification of amino acids involved in recognition by dengue virus NS3-specific, HLA-DR15-restricted cytotoxic CD4+ T-cell clones. J Virol (1996) 70:3108–17. 8627790 10.1128/jvi.70.5.3108-3117.1996 PMC190173

[71] Kurane I Zeng L Brinton MA Ennis FA. Definition of an epitope on NS3 recognized by human CD4+ cytotoxic T lymphocyte clones cross-reactive for dengue virus types 2, 3, and 4. Virology (1998) 240:169–74. 10.1006/viro.1997.8925 9454689

[72] Mathew A Kurane I Green S Stephens HA Vaughn DW Kalayanarooj S Predominance of HLA-restricted cytotoxic T-lymphocyte responses to serotype-cross-reactive epitopes on nonstructural proteins following natural secondary dengue virus infection. J Virol (1998) 72:3999–4004. 9557687 10.1128/jvi.72.5.3999-4004.1998 PMC109627

[73] Gagnon SJ Ennis FA Rothman AL. Bystander target cell lysis and cytokine production by dengue virus-specific human CD4(+) cytotoxic T-lymphocyte clones. J Virol (1999) 73:3623–9. 10196254 10.1128/jvi.73.5.3623-3629.1999 PMC104137

[74] Rothman AL Kurane I Ennis FA. Multiple specificities in the murine CD4+ and CD8+ T-cell response to dengue virus. J Virol (1996) 70:6540–6. 8794288 10.1128/jvi.70.10.6540-6546.1996 PMC190694

[75] Yauch LE Prestwood TR May MM Morar MM Zellweger RM Peters B CD4+ T cells are not required for the induction of dengue virus-specific CD8+ T cell or antibody responses but contribute to protection after vaccination. J Immunol (2010) 185:5405–16. 10.4049/jimmunol.1001709 20870934 PMC2962919

[76] Ramsbottom KM Hawkins ED Shimoni R McGrath M Chan CJ Russell SM Cutting edge: DNAX accessory molecule 1-deficient CD8+ T cells display immunological synapse defects that impair antitumor immunity. J Immunol (2014) 192:553–7. 10.4049/jimmunol.1302197 24337740

[77] Cella M Presti R Vermi W Lavender K Turnbull E Ochsenbauer-Jambor C Loss of DNAM-1 contributes to CD8+ T-cell exhaustion in chronic HIV-1 infection. Eur J Immunol (2010) 40:949–54. 10.1002/eji.200940234 20201043 PMC3031090

[78] van Leeuwen EM Remmerswaal EB Vossen MT Rowshani AT Wertheim-van Dillen PM van Lier RA Emergence of a CD4+CD28- granzyme B+, cytomegalovirus-specific T cell subset after recovery of primary cytomegalovirus infection. J Immunol (2004) 173:1834–41. 10.4049/jimmunol.173.3.1834 15265915

[79] Wang Y Bai J Li F Wang H Fu X Zhao T Characteristics of expanded CD4+CD28null T cells in patients with chronic hepatitis B. Immunol Invest (2009) 38:434–46. 10.1080/08820130902943105 19811419

[80] Akondy RS Monson ND Miller JD Edupuganti S Teuwen D Wu H The yellow fever virus vaccine induces a broad and polyfunctional human memory CD8+ T cell response. J Immunol (2009) 183:7919–30. 10.4049/jimmunol.0803903 19933869 PMC3374958

[81] Barnett ED Yellow fever: epidemiology and prevention. Clin Infect Dis (2007) 44:850–6. 10.1086/511869 17304460

[82] Sallusto F Lanzavecchia A. Heterogeneity of CD4+ memory T cells: functional modules for tailored immunity. Eur J Immunol (2009) 39:2076–82. 10.1002/eji.200939722 19672903

[83] Imai T Hieshima K Haskell C Baba M Nagira M Nishimura M Identification and molecular characterization of fractalkine receptor CX3CR1, which mediates both leukocyte migration and adhesion. Cell (1997) 91:521–30. 10.1016/S0092-8674(00)80438-9 9390561

[84] Nishimura M Umehara H Nakayama T Yoneda O Hieshima K Kakizaki M Dual functions of fractalkine/CX3C ligand 1 in trafficking of perforin+/granzyme B+ cytotoxic effector lymphocytes that are defined by CX3CR1 expression. J Immunol (2002) 168:6173–80. 10.4049/jimmunol.168.12.6173 12055230

[85] Teijaro JR Turner D Pham Q Wherry EJ Lefrancois L Farber DL. Cutting edge: tissue-retentive lung memory CD4 T cells mediate optimal protection to respiratory virus infection. J Immunol (2011) 187:5510–4. 10.4049/jimmunol.1102243 22058417 PMC3221837

[86] Iijima N Iwasaki A. T cell memory. A local macrophage chemokine network sustains protective tissue-resident memory CD4 T cells. Science (2014) 346:93–8. 10.1126/science.1257530 25170048 PMC4254703

[87] Bottcher JP Beyer M Meissner F Abdullah Z Sander J Hochst B Functional classification of memory CD8(+) T cells by CX3CR1 expression. Nat Commun (2015) 6:8306. 10.1038/ncomms9306 26404698 PMC4667439

[88] LaFleur C Granados J Vargas-Alarcon G Ruiz-Morales J Villarreal-Garza C Higuera L HLA-DR antigen frequencies in Mexican patients with dengue virus infection: HLA-DR4 as a possible genetic resistance factor for dengue hemorrhagic fever. Hum Immunol (2002) 63:1039–44. 10.1016/S0198-8859(02)00682-1 12392857

[89] Sierra B Alegre R Perez AB Garcia G Sturn-Ramirez K Obasanjo O HLA-A, -B, -C, and -DRB1 allele frequencies in Cuban individuals with antecedents of dengue 2 disease: advantages of the Cuban population for HLA studies of dengue virus infection. Hum Immunol (2007) 68:531–40. 10.1016/j.humimm.2007.03.001 17509453

[90] Malavige GN Rostron T Rohanachandra LT Jayaratne SD Fernando N De Silva AD HLA class I and class II associations in dengue viral infections in a Sri Lankan population. PLoS One (2011) 6:e20581. 10.1371/journal.pone.0020581 21694773 PMC3111420

[91] Jessie K Fong MY Devi S Lam SK Wong KT. Localization of dengue virus in naturally infected human tissues, by immunohistochemistry and in situ hybridization. J Infect Dis (2004) 189:1411–8. 10.1086/383043 15073678

[92] Durbin AP Vargas MJ Wanionek K Hammond SN Gordon A Rocha C Phenotyping of peripheral blood mononuclear cells during acute dengue illness demonstrates infection and increased activation of monocytes in severe cases compared to classic dengue fever. Virology (2008) 376:429–35. 10.1016/j.virol.2008.03.028 18452966 PMC2546568

[93] Rothman AL. Immunity to dengue virus: a tale of original antigenic sin and tropical cytokine storms. Nat Rev Immunol (2011) 11:532–43. 10.1038/nri3014 21760609

[94] Fesnak AD June CH Levine BL. Engineered T cells: the promise and challenges of cancer immunotherapy. Nat Rev Cancer (2016) 16:566–81. 10.1038/nrc.2016.97 27550819 PMC5543811

[95] Buck MD O’Sullivan D Pearce EL. T cell metabolism drives immunity. J Exp Med (2015) 212:1345–60. 10.1084/jem.20151159 26261266 PMC4548052

[96] Cham CM Driessens G O’Keefe JP Gajewski TF. Glucose deprivation inhibits multiple key gene expression events and effector functions in CD8+ T cells. Eur J Immunol (2008) 38:2438–50. 10.1002/eji.200838289 18792400 PMC3008428

